# Annotations

**Published:** 1904-06-11

**Authors:** 


### ANNOTATIONS.

### A Girgenti Home.

SOME three years ago the Corporation of Glasgow, on the suggestion of the Government, consented to establish, near the city, a Girgenti Home for inebriates. The experiment, to judge from the report recently presented to the Scottish secretary, and from a discussion in the Town Council, does not seem to have proved an unqualified success. The financial statement shows an outlay at the rate of £1 5s. 3d. per head per week, and of the 35 inmates who have passed through the Home and are out on license, less than 50 per cent. may be said "to be giving satisfaction to their guardians, and doing well." Several of the city fathers, to judge from their remarks, have come to the conclusion that the game is not worth the candle. On the other hand, the managers of the Home are by no means unhopeful. They recognise as a weak part of the scheme the loss of supervision over the inmates after these are discharged from the home, and they suggest that steps be taken to provide employment after the term of three years' residence is completed. One of the town councillors contended that the Girgenti Home at the best only obtained results by a discipline of years equal to what was possible by other methods in the course of a few months, to which it was replied that the managers of the home had appointed a committee to investigate the "gold cure" for inebriety. A remarkable feature about the Glasgow Home is the number of inmates who escape or attempt to escape from it. In 1903 the average daily number of inmates was 42·07, and no fewer than 24, in spite of the risk of three months' imprisonment or a fine of £20, made successful or unsuccessful attempts to regain their freedom. On the whole, the experience of the Glasgow Home to the present date seems to render it doubtful whether the experiment ought to be continued as a charge on the community unless either more satisfactory results can be obtained or the charges can be reduced to a more reasonable level.

### Doctors and Book-keeping.

AT the public examination last week before Mr. Registrar Brougham of a medical practitioner, who applied for an order of discharge, the Official Receiver complained that proper books of account had not been kept by the bankrupt. On behalf of the latter it was stated that his ledger showed the moneys paid and debts owing to him by his patients. There were, in addition, the banker's pass-book and a pocket-book containing a monthly summary of fees due. For the rest, it was contended by the solicitor representing the bankrupt that a general practitioner was not required to keep books like those of a trader. The Registrar subsequently expressed his opinion that the bankrupt was not called upon to keep elaborate books; and his discharge was suspended for three years, not on account of insufficient book-keeping, but because he had attempted to increase a diminishing income by hazardous speculations on the Stock Exchange. There is no doubt that a medical man is not required by law to keep books like those of a trader, neither is it any advantage to him to adopt an elaborate system of double entry. But, entirely apart from any legal question, it is of the utmost importance that books which show the amounts he receives from his patients during the year, his annual expenditure in the pursuit of his profession, and the fees which are still due to him, should be posted up in the most precise and accurate manner. We are afraid, however, that this is not by any means as generally recognised as it should be, and that there are many doctors who keep their accounts so loosely that it would be difficult for them, at a given moment, to state how they stand. Some of them only send in bills at irregular intervals, and then are surprised that their patients think the charges heavy. In his own interest a doctor should be a man of business. It saves him trouble in the end in any case; and if he desires to dispose of his practice it is obvious that accurately kept books which will disclose almost at a glance its financial value, must greatly facilitate negotiations. There was a time when it was considered to be beneath the dignity of a professional man to trouble his head about business details, but that pernicious and snobbish idea has long since been exploded, and a doctor's books of account should enable him, if he chooses, to make annual balance sheets which an auditor would pass.

### Japanese Medicine and Pharmacy.

ACCORDING to a correspondent of *The Chemist and Druggist*, medicine and pharmacy in Japan have hardly reached the level of efficiency which appears to have been attained in the art of war. Japanese druggists, we are told, are of two classes, the one certificated and qualified to compound medicines, the other dealing in drugs and sundries. The native doctors for the most part dispense their own medicines, though a few write prescriptions only, these latter for the most part having studied in Germany and holding a university degree. The metric system is used for both weights and measures. There is a considerable popular demand for household remedies on the lines familiar in England, and many of these correspond exactly with those which have here long since fallen into disuse. Thus snakes, lizards, frogs, crabs, and other animals are kept in a dried condition in the native drug stores, and enjoy more or less repute as medicinal agents. Secret remedies of various kinds are also to be obtained, and are announced as possessing the comprehensive therapeutic claims which we are accustomed to in the advertisement columns of our own daily press. Face paints and powders and various dentifrices are much in vogue, and plasters to relieve headache and neuralgia are largely employed. Massage is used in rheumatism and in nervous affections, and is to a large extent in the hands of the blind. The manufacture of mineral waters is carefully supervised by the authorities, and fines are inflicted in any case where the official standard of purity is departed from. Foreign pharmacists wishing to practise in Japan must submit their diplomas to the Government and must obtain a special license to conduct a chemist's business; without this license it is illegal to practise pharmacy in Japan. The medical and dispensing arrangements for the army are kept strictly in the hands of the military authorities.

